# Is Closed Manipulative Reduction and Percutaneous Kirschner Wiring of Supracondylar Humeral Fracture in Children as Day-Care Surgery a Safe Procedure ?

**DOI:** 10.5704/MOJ.1307.006

**Published:** 2013-07

**Authors:** Ashok R Nayak, K Natesh, Monish Bami, S Vinayak

**Affiliations:** Department of Orthopaedics, Shri B.M. Patil Medical College, Karnataka, India; Department of Orthopaedics, Shri B.M. Patil Medical College, Karnataka, India; Department of Orthopaedics, Shri B.M. Patil Medical College, Karnataka, India; Department of Orthopaedics, Shri B.M. Patil Medical College, Karnataka, India

## Abstract

Introduction:Supracondylar fracture of the humerus is a common injury in children. It accounts for 60% of fractures around the elbow children. If the fracture is not treated properly it may give rise to many complications like malunion, Volkmann’s ischemic contracture, nerve injury, arterial injury, skin slough, heterotopic bone formation , and stiffness of elbow. The management of displaced supracondylar fracture of the elbow is one of the most difficult of the many fractures seen in children. The purpose of the study was to evaluate the anatomical and functional results of treatment of supracondylar fractures of humerus with closed reduction and percutaneous ‘K’ wire fixation as a day care procedure and record associated complications, thus decreasing the cost of treating these fractures and hospitalization. Methods: Fifty displaced closed extension type supracondylar fractures (Gartland’s type III) of the humerus in children were treated by closed reduction and percutaneous fixation with Kirschner wires. All the patients selected for this study had been treated in a day care unit and were discharged in the same evening and followed up at 3 and 6 weeks and 3 months. Open fractures, fractures with neurovascular complications and children older than 15 yrs were excluded. The final results were evaluated by Flynn’s criteria. Results: The majority (72%), of the patients had fracture displaced posteomedially, Fourty one of the fifty patients had satisfactory results. The majority of the patients were male, and the average age was 8-9 years. Conclusion: Percutaneous fixation of supracondylar humerus done as a day care procedure is an acceptable modality of treatment and reduces the duration of hospital stay for the patient.

**Key Words:**

Supracondylar humerus, K-wire fixation, day care procedure

## Introduction

Supracondylar fracture is a common injury in children. It accounts for 60% of fractures about the elbow joint in children1. The rate of occurrence increases steadily in the first five years of life to peak at 5-7years of age^2^. If the fracture is not treated properly it may give rise to many complications like malunion, Volkmann's ischemic contracture, nerve injury, arterial injury, skin slough, heterotopic bone formation, and stiffness of elbow. The management of displaced supracondylar fracture of the elbow is one of the more difficult of the many fractures seen in children^3^. Closed reduction with splint or cast immobilisation has traditionally been recommended for displaced supracondylar fractures, but loss of reduction and the necessity for repeated manipulations is likely to lead to malunion producing varus or valgus deformity of elbow and elbow stiffness^4^. Traction (skin or skeletal), which has been used for many years, has been shown to be safe and reliable, but it has the drawback of requiring a long stay in the hospital^5^.

Open reduction and internal fixation have generally been reserved for specific indication mainly for an open fracture^5^, a fracture requiring vascular exploration, or an irreducible fracture^5^. Recent studies have shown good functional results with closed reduction and percutaneous fixation using K wires^6^, which is the commonly accepted treatment of displaced supracondylar fractures of the humerus in children.

The purpose of the study was to evaluate the anatomical and functional results of treatment of supracondylar fractures of humerus with closed reduction and percutaneous K-wire fixation done as a day care procedure and to record associated complications.

## Materials and Methods

Fifty displaced closed extension type of supracondylar fractures (Gartland’s type III) of the humerus in children were treated by closed reduction and percutaneous fixation with two lateral Kirschner wires. This study was conducted in Shri B. M. Patil Medical College Hospital and Research Center, Bijapur, Karnataka, on patients less than 15 years of age and diagnosed with closed supracondylar humerus fracture. Patients having open fractures, fractures associated with neurovascular complications, failed closed reductions, fractures older than three weeks and other bony injuries to ipsilateral limb were excluded from this study.

All patients selected for the study were examined according to a set protocol and relevant investigations were carried out. The fractures were classified according to Gartland’s Classification and fixed percutaneously as a day care procedure. Post-operatively the patients were placed in a well padded posterior splint with elbow flexed to 90 degrees. Immediate post-operative radiographs were taken to determine the maintenance of reduction. The patients were discharged in the evening and regular follow-ups were performed. Four weeks later the splint and pins were removed and active range of motion exercises were encouraged. A special mention and warning were given after the removal of the splint about avoiding massage and passive stretching of the elbow joint. Further follow-ups were done at three and six weeks and three months. The patients were examined clinically and with radiological assessment for range of motion and carrying angle.

The final results were evaluated by Flynn’s criteria^7^. The results were graded as excellent, good, fair and poor according to loss of range of motion and loss of carrying angle.

## Discussion

In our series, age distribution was 4 to 15 years , with an average of 8.9 years Majority of the patients i.e. 17 (34%) were from 10 – 12 years age group, followed by 15 (30%) patients in 7 - 9 years age group. Majority of the patients were males 35 (70%) and 15 (30%) females. The major causes of fracture in our study was fall while playing in 28 (56%) patients followed by fall from bicycle in 15 (30%) patients and in 7 (14%) patients was due to fall from tree. The fracture occurred more on the left side in 34 (68%) patients and on the right in 16 (32%) patients. In our study, we had 36 (72%) patients with posteromedial displacement and 14 (28%) patients with posterolateral displacement. At the final follow-up, 0-50 loss of range of motion of the affected extremity was noted in 30 patients and more than 150 loss of range of motion was noted in only one patient and mean loss of range of motion was 6.80 in our study. At the final follow-up 0-50 carrying angle loss of the affected extremity was noted in 34 patients. More than 150 carrying angle loss was noted in only one patients and mean loss of carrying angle was 5.140 degrees in our study.The final results were evaluated by Flynn’s criteria. In our study 49 patients had satisfactory results, of whom, 34 (68%) patients were rated as excellent, 10 (20%) patients good and 5 (10%) patients fair. Only one patient (2%) had unsatisfactory result, rated as poor due to loss of motion at the elbow.

We had four cases of superficial pin track infection which was treated by appropriate antibiotics. We had two cases of iatrogenic ulnar nerve palsy and with full gradual recovery within four months. These were three cases of cubitus varus deformity which was later treated by corrective osteotomy.

## Results

The aims of treatment of supracondylar fractures are to achieve functionally and cosmetically satisfactory results and to avoid complications. Assuring a low cost and decreasing the hospitalization period are important for both surgeons and patient’s parents. Traction is still an effective method of treatment but has many drawbacks. First, it is expensive. Second, when the extremity is swollen, it is very risky to attempt skin traction. Third, when skeletal traction is attempted, it poses some problems and prolongs the hospitalization period^8^. Primary open reduction and internal fixation is an alternative method of treatment. There are several different surgical approaches to the fracture site. The most heavily criticised has been the posterior approach which is claimed to be the method most likely to cause loss of elbow movement, and infection^9^. Because of this problem, the major indications for a primary open reduction include an open fracture, failure to achieve an adequate closed reduction or vascular compromise that worsens especially with the manipulative technique^9^. Hence closed reduction and percutaneous pinning have become a popular method recently. The American Academy of Orthopedic Surgeons in their recent published guidelines for the management of supracondylar fracture in children have recommend that CRPP be done within 8-12 hours if there is no neurovascular compromise, tenting of the skin or worsening edema^10^. The present study was conducted to assess the results of closed reduction and percutaneous fixation with Kirschner wires for displaced extension type of supracondylar fractures of the humerus in children.

As per Flynn’s Criteria, the final results in our study were excellent in 34(68%) patients, good in 10 (20%) of patients, fair in 5(10%) of the patients and poor in one (2%) patient. These results were comparable to other studies conducted around the world on different modalities of treatment of fractures of supracondylar humerus in children as shown in Table I.

In our study all fractures united around four weeks. We had 4 cases of superficial pin track infection which were treated by appropriate antibiotics. We had two cases of iatrogenic ulnar nerve palsy resulting from the medial pin due to improper pin insertion or stretch of the ulnar nerve over the medial pin. They showed progressive improvement in time and regained full neurologic function within four months. We had three cases with cubitusvarus deformity which require corrective osteotomy later. No patient had pain or symptoms related to the elbow.

Boyd et al preferred two parallel laterally inserted K-wires for percutaneous fixation, if fracture is stable. If it is unstable, they prefer crossed medial and lateral K- wires. In their series, 70 of 71 patients had satisfactory results. Six patients had neurovascular complications. One Ulnar and two interosseus nerve palsies were documented before surgery, and two cases treated with crossed medial and lateral pins had iatrogenic ulnar nerve palsies at postoperative clinical examinations. All nerve palsies had completely recovered by the time of follow up evaluation^10^. The mean loss of range of motion was 6.8 degrees in the present study. Nacht JL et al., noted mean loss of range of motion was 7.8 degrees at the final follow up in their study^11^.

The mean loss of carrying angle was 5.14 degrees in our study. Flynn JC. et al., reported mean loss of carrying angle of 6.2 degrees^6^.

Pirone et al reviewed 230 patients treated by different methods. Highest percentage of excellent results was achieved by percutaneous Krischner wire fixation (78%), skeletal traction (67%) and open reduction with internal fixation (67%). The ulnar nerve was injured due to the medial pin and two pin track infections which had occurred in the percutaneous pin fixation group^5^.

The result of the present study compare favourably with those of other previously reported methods of treatment of the displaced supracondylar fractures of the humerus in children.

**Table I:**
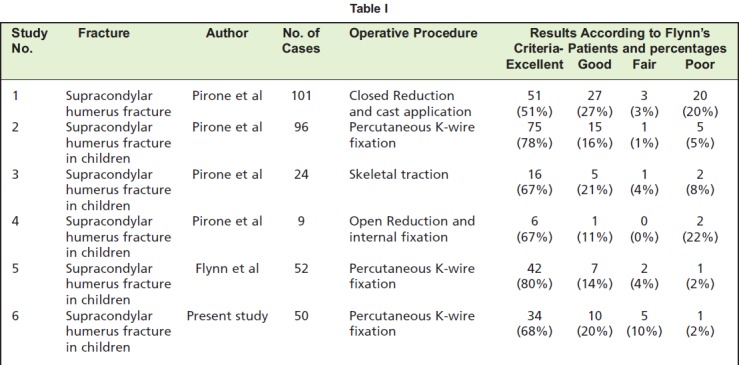


**Fig. 1:**
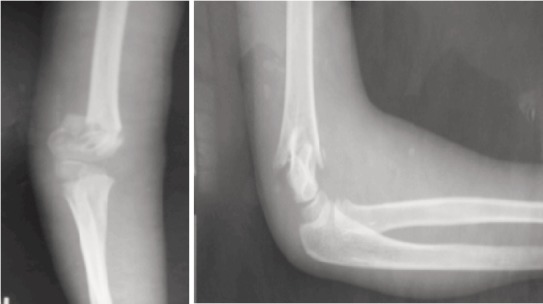
: Pre-Reduction X-rays AP and Lateral view of the patient.

**Fig. 2:**
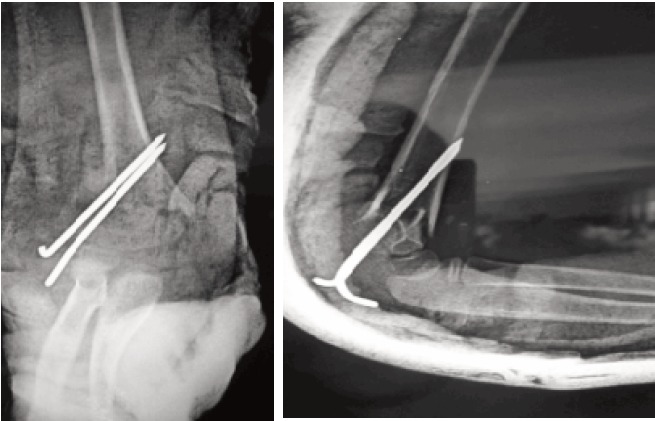
: Immediate Post- operative radiograph in AP and Lateral view.

**Fig. 3:**
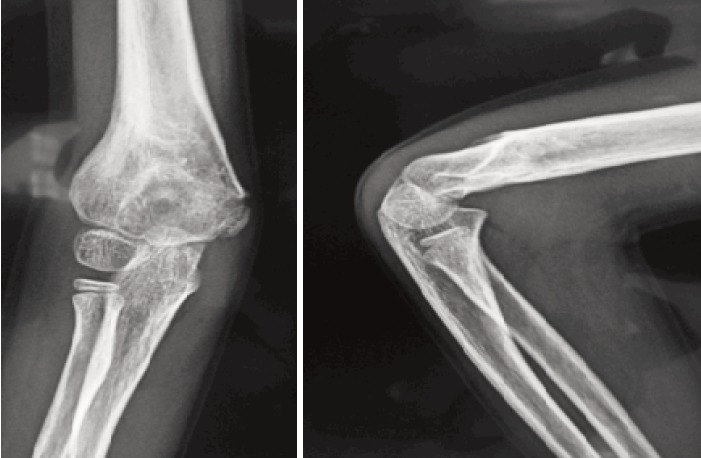
: Final Follow-up radiographs of the patient.

## Conclusion

Percutaneous wire fixation done as a day care procedure in supracondylar humerus fractures in the paediatric age group is a safe and effective procedure for fracture fixation with good functional and cosmetic outcome for the patient with minimal hospital stay.

No problems developed in our patients from the brief stay in the hospital following the reduction and stabilization of the fractures with percutaneous pins. The few problems that developed in our patients were the result of errors in the surgical technique and not related to the short stay. The stability of the fracture and any associated complications should be evaluated on regular follow-ups by clinical evaluation and radiograph as an when necessary.
